# The effects of nonsuicidal self-injury on parenting behaviors: a longitudinal analyses of the perspective of the parent

**DOI:** 10.1186/s13034-015-0059-2

**Published:** 2015-07-08

**Authors:** Imke Baetens, Laurence Claes, Patrick Onghena, Hans Grietens, Karla Van Leeuwen, Ciska Pieters, Jan R. Wiersema, James W. Griffith

**Affiliations:** KU, Leuven, Belgium; University of Groningen, Groningen, The Netherlands; Ghent University, Ghent, Belgium; Northwestern University, Evanston, IL USA

**Keywords:** Nonsuicidal self-injury, Adolescence, Consequences, Parent-reported data, Parenting behaviours, Longitudinal, Cross-lagged analyses

## Abstract

**Background:**

The present study is the first to examine predictors and consequences of nonsuicidal self-injury (NSSI) in adolescence using parent-reported data in a longitudinal design. Across three time points, we examined the reciprocal effects of parent-reported parenting behaviors as they are related to adolescents’ NSSI.

**Methods:**

The present study is a three-wave prospective study in a large sample of community adolescents and their parents. At time 1 (age 12), the sample consisted of 1396 adolescent reports and 1438 parent reports. Time 2 (age 13) included 827 adolescent and 936 parent reports. At time 3 (age 14), 754 adolescent and 790 parent reports were obtained. Engagement in NSSI (adolescent report) was determined by an affirmative response to the item ‘Have you intentionally injured yourself (e.g., cut, burn, scratch) this year, without the intent to die?’. Parental awareness of NSSI at age 13 and 14 was examined using a single-item screening question. Parenting behaviors were examined by the parent versions of the Parental Behavior Scale.

**Results:**

Results showed that although NSSI was reported by 10 % of the adolescents, only 3 % of the parents were aware of the NSSI behaviors of their children. Cross-lagged analyses showed a reciprocal relationship between NSSI and parenting behaviors over time. We found a significant effect of both positive parenting and controlling parenting on the presence of NSSI at time 2. But vice versa NSSI also has an effect on parenting behaviors over time. Results showed that NSSI at time 1 has an impact on controlling parenting behaviors, namely punishment at time 2. NSSI at time 2 showed an impact on parent’s perception of positive parenting, parental rule setting, punishment and harsh punishment.

**Conclusions:**

The present study examined predictors and consequences of NSSI in a longitudinal design, and emphasized the importance of examining reciprocal interactions between NSSI and parenting behaviors. Furthermore, it is the first study to examine parent-reported data in a longitudinal design and gives insight into parents’ perspectives.

## Background

Nonsuicidal self-injury (NSSI) is defined as socially unacceptable, intentional, and direct injuring of one’s own body tissue *without* suicidal intent [1]. In community samples of young adolescents between the ages of 11–15, the lifetime prevalence of NSSI is estimated to be 7-11 % [2–5], with an increase in the prevalence of NSSI behaviors between the ages 13–15, and a decrease from 16 years onwards (for a review see [6]). The present study focuses on adolescents between ages 13 through 15, where NSSI behavior shows a sharp and troubling upturn.

**Table 1:** Correlation matrix

	1	2	3	4	5	6	7	8	9	10	11	12
1. SOGpP1	1											
2.SOGpR1	.373^**^	1										
3.SOGpS1	.021	.247^**^	1									
4.SOGpH1	-.210^**^	-.037	.188^**^	1								
5.SOGpP2	.678^**^	.264^**^	-.039	-.217^**^	1							
6.SOGpR2	.337^**^	.500^**^	.088^**^	-.088^**^	.438^**^	1						
7.SOGpS2	.021	.187^**^	.637^**^	.150^**^	.008	.144^**^	1					
8.SOGpH2	-.159^**^	-.009	.197^**^	.511^**^	-.200^**^	-.127^**^	.199^**^	1				
9.SOGpP3	.662^**^	.263^**^	.023	-.121^**^	.706^**^	.326^**^	.004	-.109^**^	1			
10.SOGpR3	.333^**^	.526^**^	.138^**^	-.005	.374^**^	.668^**^	.146^**^	-.043	.453^**^	1		
11.SOGpS3	.041	.212^**^	.548^**^	.163^**^	-.007	.145^**^	.645^**^	.145^**^	.063	.247^**^	1	
12.SOGpH3	-.052	.047	.148^**^	.467^**^	-.094^*^	.050	.127^**^	.483^**^	-.085^*^	.005	.208^**^	1

*Note*. The full model is shown in this figure. *SOGpp* = Parent-reported positive parenting; *SOGpr* = Parent-reported rules; *SOGps* = Parent-reported punishment; *SOGph* = Parent-reported harsh punishment. The numbers at the end of the variable names present indicators of time points 1. 2 or 3

*p < .05. **p < .01. ***p < .001

Besides the well-studied intrapersonal predictors of NSSI (e.g., general psychological distress, previous NSSI behaviors, etc. – see review [6]), several interpersonal predictors are shown to play an important role in the onset and maintenance of NSSI behaviors. For example, parenting has been identified as an important predictor for NSSI in community samples of adolescents. Positive parenting (i.e., parenting characterized by warmth and support) is associated with less frequent NSSI [7]. High controlling parenting behaviors (i.e., parenting behaviors wherein a parent wishes to influence the behavior and psychological world of the adolescent) are associated with more frequent NSSI [8]. In their review, Plener and colleagues [6] found three studies that examined family-related predictors of NSSI in a longitudinal design. Results showed significant influences of onset of parental depression, lower perceived family support and perceived problems with parents. Although research on predictors is relevant to develop interventions for NSSI, current research and clinical practice lack insight into the consequences of NSSI on family functioning. Qualitative research shows that NSSI has a large impact on parents and family life [9–11]. For example, as a consequence of NSSI, parents become hyper-vigilant about adolescent’s well-being, increase monitoring their child’s emotional state, and increase control and parental rule setting. Some parents also report changing conflict management after NSSI. Specifically, they try to avoid conflict with the adolescent in case the conflict triggers another episode of NSSI. Most research results on consequences of NSSI on family functioning are based on qualitative data [9]. To date, only two quantitative studies have examined the consequences of NSSI on the family. Hilt et al. [4] found support for a social reinforcement mechanism, where the quality of relationship with fathers increased after NSSI. On the other hand, Baetens and colleagues [2] found that adolescents who engage in NSSI change in their perception of parenting behaviors over time: Adolescents who self-injure perceive a significant increase in monitoring and rules, irrespective of whether parents are aware of NSSI acts.

Notably, most quantitative research examining predictors and consequences of NSSI rely on adolescent-reported data. Negative cognitive biases might modify the perception of family functioning in time of distress, so non-self-report research is also needed. To address this gap in the literature [2], the present study examines the predictors and consequences of NSSI as reported by parents.

### Aims of the study

Given that NSSI is often secretive [12], the first aim of the present study was to investigate parental awareness of their children’s NSSI behaviors. We aimed to compare prevalence rates of self-reported NSSI to parent reports. The second aim of the present study was to examine antecedents and consequences of NSSI in relation to parenting behaviors. In order to examine sequential changes over time, we performed cross-lagged analyses. We hypothesized that NSSI will be predicted by more controlling and less supportive parenting behaviors at preceding time points. Consistent with Baetens and colleagues [2], we hypothesized that adolescents’ NSSI will alter parenting behaviors one year later.

## Methods

### Procedure and participants

All respondents included in this study participated in the prospective cohort study JOnG! [13], which followed the development of mental health, family and healthcare of a Flemish cohort of twelve-year olds. All parents of twelve-year old adolescents living in eight districts (both urban and rural areas) of Flanders (*N* = 9861) were invited to participate in this study. This sample represents 15.2 % of all twelve-year olds in Flanders [13]. The JOnG!-study is commissioned, financed and steered by the Ministry of the Flemish Community (Department of Economics, Science and Innovation; Department of Welfare, Public Health and the Family). The work was performed by the Policy Research Centre for Welfare, Public Health and the Family and in assistance of a collaboration between two Flemish universities.

The JOnG! study was approved by the Ethics Committee of both universities cooperating in the JOnG! project. All participating adolescents and parents gave informed consent. In addition, parents gave informed consent for the adolescent reports.

In total, 1499 families provided informed consent and agreed to participate in this longitudinal study. Of all 1499 families who provided informed consent, we received 93.20 % (*N* = 1397) valid adolescent reports (age 12) and 95.93 % (*N* = 1438) valid parent reports (i.e., valid reports are those questionnaires with less than 10 % missing data). Parent reports were completed by 88.70 % mothers, 4.30 % fathers, 1.20 % step, adoptive or foster parents. Adolescent reports consisted of 54.70 % girls and 45.30 % boys. At time 2, 1132 adolescents (age 13) and their parents participated in this study resulting in 827 valid adolescent reports (73.57 %) and 936 parent reports (82.68 %). At time 3 (one and a halve years after time 2), in total 839 adolescents (age 14) and their parents participated, resulting in 754 valid adolescent reports (89.86 %) and 790 valid parent reports (94.50 %). The adolescent-reported data were described elsewhere [2]; whereas the present study focuses on the parent-reported data.

Participants (both adolescents and parents) with and without complete data were compared in terms of gender, district, presence of psychiatric disorder, and psychological complaints. The Missing Completely At Random (MCAR) [14] test resulted in a non-significant Chi-square value, *χ*^2^ (177) = 175.70, which suggests that missing data are completely at random. Missing data are handled using full information maximum likelihood (FIML). Data analyses were conducted with Mplus using a robust mean- and variance-adjusted chi square estimator (WLSMV), which is appropriate for binary variables, in this study NSSI (present/absent) [15]. NSSI is a binary predictor (0/1), which only changes from 0 to 1 and not by a standard deviation. Using two different methods of standardization in one figure would be confusing, therefore only the unstandardized pathways are shown in the figure.

### Measures

Engagement in NSSI was assessed by means of a single-item screening question in both adolescent and parent questionnaires. Adolescents were asked at time 1 ‘Have you ever intentionally injured yourself (e.g., cut, burned, scratched), without the intent to die? (Yes/No)’, and at time 2 and time 3 they were asked “Did you intentionally injure yourself since the previous survey? (Yes/No)’. Parent reported NSSI at age 13 and age 14 was examined as follows: ‘Has your son/daughter ever intentionally injured him/herself – e.g., by cutting, burning, scratching – without the intent to die?’ (Yes/No). According to Muehlenkamp and colleagues [5], the use of a single-item measure of NSSI renders consistent estimates of NSSI prevalence.

Parenting behavior was measured with the parent-reported Parental Behavior Scale, short version (PBS) [16] (time 1, 2 and 3). The subscales ‘positive parenting’, ‘parental rules setting’, ‘punishing’, and ‘harsh punishing’ were used in this study. The PBS subscales ‘positive parenting’ and ‘parental rules setting’ (including both limit setting and learning rules) were used as indicators of parental support. The PBS subscales ‘punishment’ and ‘harsh punishment’ were used as indicators of parental control. The results of confirmatory factor analyses confirmed this model in previous studies [2]. At time 1, the Cronbach’s alpha coefficient was .85 for positive parenting and .70 for parental control. The Cronbach’s alpha coefficients for positive parenting and parental control were respectively.86 and .78 at time 2. At time 3, the Cronbach’s alpha coefficients were.87 for positive parenting and .77 for parental control.

### Analyses

First, means, standard deviations, correlations, and reliability coefficients were calculated (see Table 1). All continuous subscales were rescaled to Percent of Maximum Possible Scores (POMP) [17]. A POMP score is the percentage of the distance (0-100 %) from the minimum to the maximum of a scale, which allowed us to examine both the magnitude and impact of the observed relationships between variables even when the underlying units of metric are different.

**Fig. 1:**
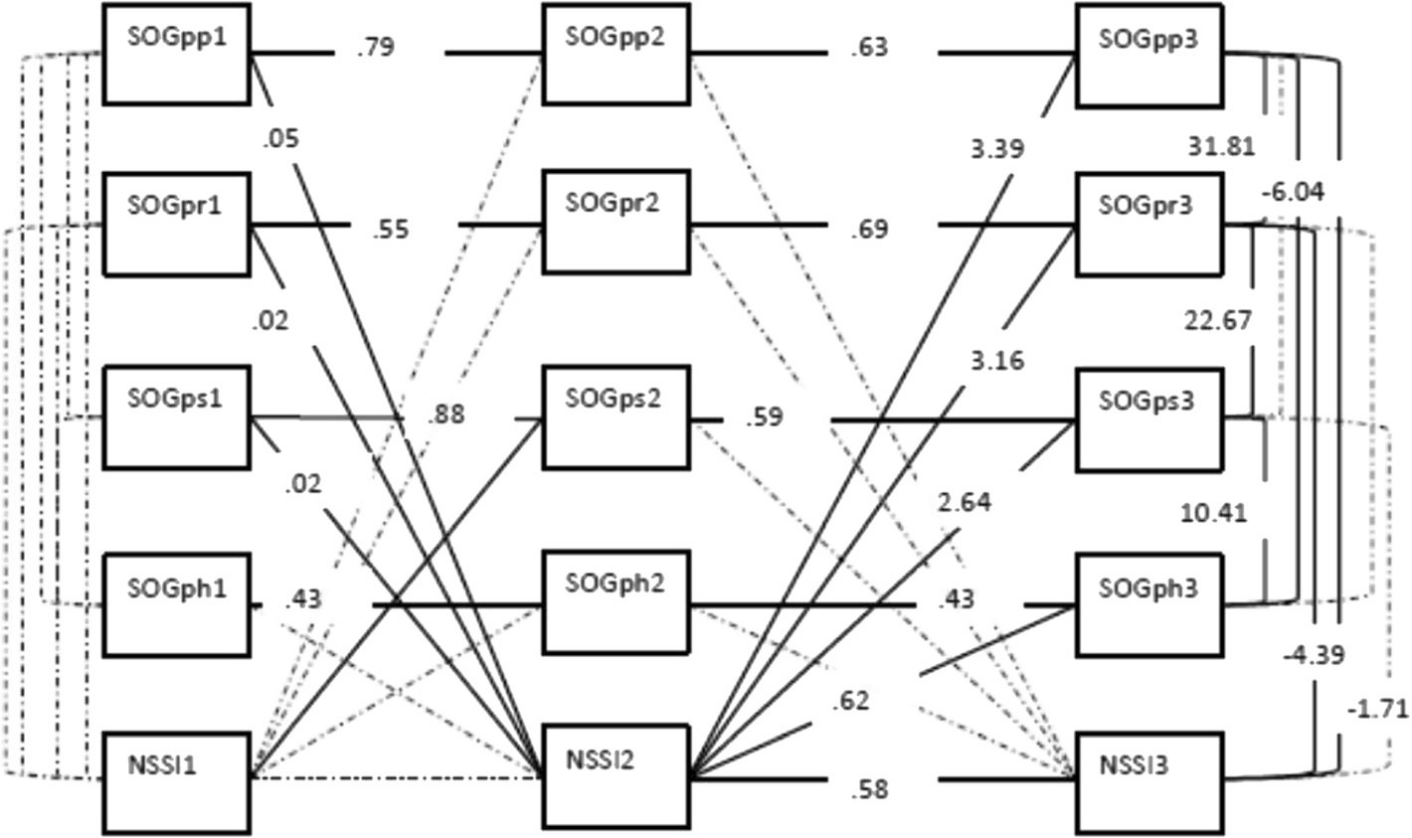
Cross-lagged path model: Reciprocal effects between adolescent reported NSSI and parent-reported parenting behaviors at three time points. *Note*. The full model is shown in this figure. *SOGpp* = Parent-reported positive parenting; *SOGpr* = Parent-reported rules; *SOGps* = Parent-reported punishment; *SOGph* = Parent-reported harsh punishment. The numbers at the end of the variable names present indicators of time points 1, 2 or 3. Dotted lines are non-significant paths. The unstandardized estimates are shown in the figure for the significant paths

Cross-lagged path analyses were conducted using Mplus 7.3 [15]. The four subscales of parent-reported PBS [16] at all three time points, next to adolescent reported NSSI behaviors at three time points were entered in a cross-lagged model to examine reciprocal effects. The full model is shown in Fig. 1. Model fit was estimated by means of the Comparative Fit Index (CFI) and the Root Mean Square Error of Approximation (RMSEA). The CFI should exceed .90 for a reasonable fit and .95 for a good fit to the data, and the RMSEA should be less than .05 for a close approximate fit, or between .05 and .08 for a reasonable fit to the data [18].

## Results

### Aim 1: NSSI prevalence

At age 12, 5.15 % (72/1397) of the adolescents reported having engaged in NSSI. One year later (at age 13), 2.78 % (23/827) of the adolescents reported having engaged in NSSI in the past year. At age 14, 5.13 % (40/754) adolescents answered positive on the NSSI screening question. The lifetime prevalence of NSSI as reported by adolescents was examined using 533 valid adolescent-reports, which participated in all three time points. In total, 10.70 % of the adolescents reported having engaged in NSSI at least once before the age of 15.

Parental awareness of NSSI was examined at time 2 and time 3. At time 2, 1.52 % (14/921^a^) of the parents reported NSSI behavior by their son or daughter. Parent-reported NSSI was significantly associated with adolescent-reported NSSI, *χ*^*2*^ (1, *N* = 788) = 51.12, *p* < .001; Φ = .26. At time 3, 3.18 % (25/754^b^) of the parents reported that their son/daughter had ever engaged in NSSI. Also at time 3, parent-reported NSSI was significantly associated with adolescent-reported NSSI, *χ*^*2*^ (1, *N* = 733) = 166.02, *p* < .001; Φ = .48. Comparing parent-reported lifetime prevalence with adolescent-reported lifetime prevalence, results showed that one in three parents knew that their sons/daughters are engaging in NSSI behaviors. Of all parents who reported NSSI, 86.21 % indicated they have talked about NSSI with their son/daughter who has been engaging in NSSI.

### Aim 2: Cross-lagged analyses on the relationship between NSSI and parent-reported parenting behaviors on three time points

In a first step, the model fit is compared to the aforementioned model fit indices, as described by Kline [18]. The CFI was 0.908, which we considered a reasonable fit. Also the RMSEA indicated an adequate fit; RMSEA = 0.079, 90 % CI [0.072-0.086].

Figure 1 displays all significant reciprocal effects between adolescent-reported NSSI and parent-reported parenting behaviors (positive parenting, parental rule setting, punishment and harsh punishment). Non-significant paths are dotted. Previous NSSI (at time 2) was significantly associated with NSSI at time 3. NSSI at time 1 was not significantly associated with NSSI at time 2. Focusing on antecedents, results showed that parent-reported supportive parenting behaviors (all subscales, except for harsh punishment) (at time 1) have a significant effect on NSSI at time 2. Results also revealed that the presence of NSSI at time 1 is significantly related to an increase in parent-reported punishment at time 2. Focusing on consequences, results showed that NSSI at time 2 is significantly related to parent-reported parenting behaviors at time 3. The valence of this relationship was positive, meaning that when NSSI is present at time 2, parents tend to report more supportive parenting behaviors (positive parenting and monitoring) and more controlling behaviors (punishment and harsh punishment) at time 3.

Furthermore, the results of the cross-lagged analysis also showed a negative association between NSSI at time 3 and positive parenting behaviors at time 3. When adolescents reported NSSI at time 3, parents tended to report less positive parenting practices and less parental rule setting at time 3.

## Discussion

The present study is the first prospective study on adolescents’ NSSI using parent-reported data. The first research aim of this study was to examine parental awareness of NSSI behaviors in a large community sample of adolescents. The life-time NSSI prevalence, reported by adolescents, is slightly higher than the prevalence rates in young adolescence in previous studies [3, 4], with a total prevalence of 10.70 % at three time points. Comparing parent-reported lifetime prevalence of NSSI with adolescent-reported lifetime prevalence, shows that approximately one in three parents knows that their son/daughter is engaging in NSSI.

Secondly, the results of the cross-lagged analysis confirm a reciprocal effect between NSSI and parenting behaviors. Parenting behaviors are related with NSSI as antecedents over time, whereas NSSI also has a significant effect on parenting over time. Focusing on the effect of parenting behaviors on NSSI, results of the current study show that both positive and controlling parenting are associated with NSSI over time. The positive relationship between NSSI and controlling parenting is in line with previous research [8]. Inconsistent with previous findings [6], the cross-lagged analysis shows a positive relationship between NSSI and supportive parenting. This might be explained by the fact that the current study is the first study examining parents’ perspectives, and previous self-reported studies might be affected by negative cognitive biases of adolescents in distress (i.e., negative interpretations of their context which are congruent to negative self-esteem and negative thoughts).

Focusing on consequences of NSSI, results suggest that NSSI may evoke controlling behaviors by parents over time. Furthermore, parents of adolescents who self-injure report a positive relationship between NSSI at time 2 and supportive parenting behaviors at time 3: parents are supportive and try to help their child by increasing support and monitoring. The international research is unclear whether the increase in supportive parenting is protective against future NSSI (as Tatnell and colleagues [7] suggest), or might be seen in light of a social reinforcement mechanism as Hilt and colleagues [4] suggest (which involves an increase of the adolescent problem behavior due to social attention). Longitudinal research with multiple fixed timepoints (more than 3) should examine these mechanisms in future research. Interestingly, results show a different pattern when investigating the relationship between NSSI and parenting cross-sectionally at age 14. Here the effect is negative, meaning that NSSI at age 14 is related to less supportive parenting behaviors. This result can be understood in the context of acute (family) crisis. As indicated in qualitative research [9], in times of acute NSSI, parents are often in shock and are overwhelmed by guilt and fear. Initially, they tend to react less supportive. The understanding, accepting and dealing with self-injury is usually an ongoing gradual process [10]. To fully understand the reciprocal nature between NSSI and parenting, future research should examine more complex dyadic models between NSSI and parenting behaviors, with frequent repeated measures (e.g., diary studies or 3 monthly follow-up) and multi-informant data (e.g., comparing adolescent, sibling and parent reports). Future research may also wish to collect information on frequency and severity of the NSSI as it might have an impact on parental knowledge of the behavior and how they may respond. Also, the family constellation and the number of children in the family may also play a role in the current findings, which should be explored in future studies.

The present study is the first to examine parents’ perceptions of parenting behaviors in relation to NSSI, and presents insight in the reciprocal nature between adolescent NSSI and parenting. Notwithstanding the meaningful results of this study for the international research field and clinicians worldwide, it deals with some limitations that need to be addressed in future research. First, we relied on parent-reports of parenting behaviors, which might result in biases in the data through social desirability. Parent-reported questionnaires reflecting parenting practices may contain informant-specific error, such as fake-good behavior or socially desirable answer tendencies [19]. Second, the sample is mainly comprised of mothers as parent respondents. The findings may not generalize to fathers, who may have different types of relationships with their children. Future research can examine the differences between fathers and mothers. Third, the present study was conducted in a non-clinical sample, which does not allow drawing conclusions with regard to clinical samples of adolescents. Future research should test whether the significant predictors, consequences and correlations found in the present study, could also be observed in a clinical sample of adolescents with NSSI. Fourth, although the sample size was large (*N* = 1443), the initial response rate was low (15 %). Nonetheless, previous studies [20] showed that the JOnG! study is a fair representation of the Flemish adolescents in this age group, with respect to ethnic origin and multiple indicators of socioeconomic status (i.e., educational level and employment of parents, and family income). Finally, findings may not generalize beyond the geographic area from which data were collected as parenting practices may differ culturally.

## Conclusion

The present study adds to previous research on NSSI in adolescence by examining the perception of parents. This prospective study of parent-reported data found that parents of adolescents with NSSI, who already attain higher scores on parental control and positive parenting strategies (such as providing support), tend to report similar patterns of parenting behaviors over time. Nonetheless, during time of active NSSI crisis, the whole family system might be in distress and less supportive parenting behaviors are reported. This might reflect an underlying circular feedback loop, which increases the risk for the continuation of NSSI.

## Endnotes

^a^In total, 15 parents did not answer the NSSI screening question at time 2.

^b^Parent-data on NSSI screening question were missing at time 3 for 36 parents.

## Abbreviations

NSSI: Nonsuicidal self-injury
SOGpp: Parent-reported positive parenting
SOGpr: Parent-reported rules
SOGps: Parent-reported punishment
SOGph: Parent-reported harsh punishment
